# Humanistic nursing curriculum needs: a cross-sectional survey in Shenzhen, China

**DOI:** 10.3389/fmed.2025.1654340

**Published:** 2025-11-03

**Authors:** Yurong Jiang, Yuan Gao, Shanshan Li, Haiyan Zhong, Hulu Xia, Lian Lin, Qi Zhou, Yan Liu

**Affiliations:** ^1^Department of General Practice, Shenzhen People’s Hospital (The First Affiliated Hospital, Southern University of Science and Technology; The Second Clinical Medical College, Jinan University), Shenzhen, China; ^2^Department of Vascular and Hand Surgery, Shenzhen People’s Hospital (The First Affiliated Hospital, Southern University of Science and Technology; The Second Clinical Medical College, Jinan University), Shenzhen, China; ^3^Department of Pediatrics, Shenzhen Second People’s Hospital, Shenzhen, Guangdong, China; ^4^Department of Nephrology, Center of Kidney and Urology, The Seventh Affiliated Hospital, Sun Yat-sen University, Shenzhen, China; ^5^Department of Cardiovascular Center, The People’s Hospital of Baoan, Shenzhen, China; ^6^Department of Oral and Maxillofacial Surgery, Shenzhen People’s Hospital (The First Affiliated Hospital, Southern University of Science and Technology; The Second Clinical Medical College, Jinan University), Shenzhen, China

**Keywords:** nursing education, patient-centered care, humanism, professional training and curriculum development, hospital

## Abstract

**Background:**

This study aimed to assess the training needs of nursing professionals for developing a structured humanistic nursing curriculum, emphasizing practical and theoretical components.

**Methods:**

A cross-sectional survey was conducted from January to June 2024 at Shenzhen People’s Hospital, involving 486 nursing staff members. The survey collected demographic data and training preferences across six modules: Humanistic Management, Literacy, Practice, Innovation, Care, and Education. Preferred training methods and course structures were also evaluated. Descriptive statistics were used to analyze participant responses.

**Results:**

Among participants, 89.3% were female, with 55.35% aged 25–35 years, and 85.39% holding a bachelor’s degree. The highest demand was observed for Humanistic Management (87.04%) and Humanistic Literacy (86.42%). Courses on listening skills (94.76%) and the connotation of humanistic nursing (89.6%) ranked highest. Most participants (46.3%) preferred a training model with two-thirds practical learning, and online courses (79.01%) and workshops (75.93%) were the most favored methods.

**Conclusion:**

The findings highlight the need for a structured humanistic nursing training program with an emphasis on interactive, practice-oriented learning. Future curricula should integrate emotional regulation, communication skills, and humanistic innovation to enhance patient-centered care. Further research is recommended to evaluate the long-term impact of such training on nursing practice and healthcare outcomes. Because data were collected through convenience sampling from a single tertiary hospital, the findings are not generalizable to broader nursing populations.

## Introduction

Humanities in healthcare is an interdisciplinary field that integrates humanistic principles—such as empathy, ethics, communication, and cultural sensitivity—into medical practice (1). In nursing, the application of these principles is essential in delivering patient-centered care that goes beyond physical treatment to address emotional, psychological, and social aspects of health (2). As healthcare systems worldwide strive to improve quality of care and patient outcomes, integrating humanities into nursing practice has become increasingly recognized as critical, particularly in the post-COVID era (3). It encourages a deeper understanding of patients’ needs, not just from a clinical perspective, but also by considering their emotional, personal, and cultural contexts (4). Studies have shown that patients who receive care rooted in humanistic values report higher satisfaction with their care, better adherence to treatment plans, and improved health outcomes (5, 6). Moreover, research highlights that a structured approach to person-centered care within health policies can enhance patient safety, reducing medical errors and improving care coordination. The research by the National Academies of Sciences, Engineering, and Medicine (NASEM) and from Ethiopia found that nursing programs that incorporate humanities-based curricula produce nurses who are more skilled in delivering compassionate care, which directly contributes to better patient recovery and well-being (7, 8). In addition, integrating humanities into nursing training helps address the growing concerns of burnout and stress among healthcare professionals in Pakistan (9). Nurses working in environments that prioritize empathy and emotional intelligence are better equipped to manage the emotional challenges of caregiving (10, 11). This approach not only improves job satisfaction but also enhances staff retention, which is particularly important in the face of global nursing shortages (12). The World Health Organization underscores that promoting emotional well-being among healthcare workers improves performance, reduces turnover, and strengthens overall care quality (13). The COVID-19 pandemic further amplified the urgency of humanistic nursing, particularly in China (14), as frontline nurses faced unprecedented stress while patients experienced isolation and fear (15). This crisis highlighted the critical need for care models that combine clinical expertise with empathy, emotional intelligence, and holistic attention to patient well-being (16). To assess the demand for a structured, policy-integrated nursing humanities training system, this survey aims to collect quantitative data on the specific needs and preferences of nursing staff regarding humanistic care training. The survey will explore key areas such as the core components of person-centered nursing, preferred training modules (e.g., humanistic management, literacy, and practice), and the perceived impact of such training on patient safety, health outcomes, and professional development. By identifying gaps in existing programs, this study aims to inform the design of competency-based, evidence-driven curricula that enhance both patient outcomes and nurse retention. Ultimately, humanistic nursing is a pressing priority due to rising patient expectations for holistic care, heightened emotional demands on healthcare workers, and global nursing shortages. By clarifying specific training needs and course preferences, the findings will guide policymakers, educators, and institutional leaders in developing robust, practically applicable programs that strengthen compassionate, high-quality healthcare delivery.

## Methods

The study was approved by the ethics committee of Shenzhen People’s Hospital (Approval No: 2023-EC-123). All Participants were informed about the purpose of the study, their right to withdraw at any time, and the measures taken to protect their anonymity and confidentiality. Informed consent was obtained prior to participation, with all responses being anonymous.

### Study design and participants

This cross-sectional study aimed to assess the needs and expectations of nursing professionals regarding a systematized and standardized humanistic nursing training framework at Shenzhen People’s Hospital. A total of 486 valid responses were analyzed to gain a comprehensive understanding of the demographic distribution and training needs among nursing staff. Participants were recruited through convenience sampling. The data were collected via an anonymous, self-administered electronic questionnaire. The survey was distributed between June 1, 2024, and June 30, 2024, ensuring a four-week data collection period. Inclusion criteria: All nursing staff members employed at Shenzhen People’s Hospital for a minimum of 6 months. Exclusion criteria: Temporary or contract-based nurses with less than 6 months of work experience were excluded. The sample size was determined based on available data and the convenience sampling method. However, the study did not include a power analysis to calculate the required sample size. It is recommended that future studies should include power analysis, using statistical software such as G*Power or SPSS, to ensure that the sample size is adequate to detect significant effects with sufficient power.

### Sampling method

The study used convenience sampling, where participants were selected from nursing staff at Shenzhen People’s Hospital. While this method ensured ease of access, it limits the generalizability of the findings. The study was restricted to nurses from a single hospital, and thus, data should be interpreted with caution in relation to other hospitals or regions.

### Data collection

The survey questionnaire was developed specifically for this study to collect both demographic information and nursing staff preferences regarding humanistic nursing training. It comprised several key sections: demographic data (including gender, age, education level, professional title, and years of experience); training module preferences (covering Humanistic Management, Humanistic Literacy, Humanistic Practice, Humanistic Innovation, Humanistic Care, and Humanistic Education); and preferred training methods (such as online courses, workshops, and practical hands-on sessions). To ensure clarity and relevance, a pilot study was conducted prior to the main data collection. Feedback from this pilot phase was used to refine the questionnaire items. The final survey instrument demonstrated acceptable reliability, with a Cronbach’s alpha coefficient of 0.85, indicating good internal consistency.

### Survey development and reliability testing

*Content Validity:* Expert consultations were sought to ensure the content validity of the survey items. *Reliability:* A reliability test (Cronbach’s alpha) was conducted to ensure internal consistency of the questionnaire, resulting in a coefficient of 0.85. The survey was distributed electronically via a hospital-approved platform to all nursing staff, and all responses were anonymous. Only completed and valid questionnaires were included in the final analysis.

### Data analysis

Data were analyzed using SPSS Version 26.0 (IBM Corp, Armonk, NY, United States). Descriptive statistics (e.g., mean, standard deviation, frequency, and percentage) were used to summarize demographic characteristics and training preferences. Chi-square tests and independent-samples *t*-tests were conducted to compare training needs across different demographic groups (e.g., gender, age, professional title). Statistical significance was set at *p* < 0.05. The analysis focused on identifying priority areas for training development and exploring trends across various demographic factors. The data collection process took place over 4 weeks, from June 1, 2023, to June 30, 2023. The stages of data collection were as follows: Survey Distribution: June 1, 2023 Survey Completion Deadline: June 30, 2023 Data Cleaning and Preparation for Analysis: July 1–3, 2023. This timeline ensured that responses were gathered promptly, and data analysis commenced immediately after the collection period.

## Results

### Demographic insights

**Figure 1:**
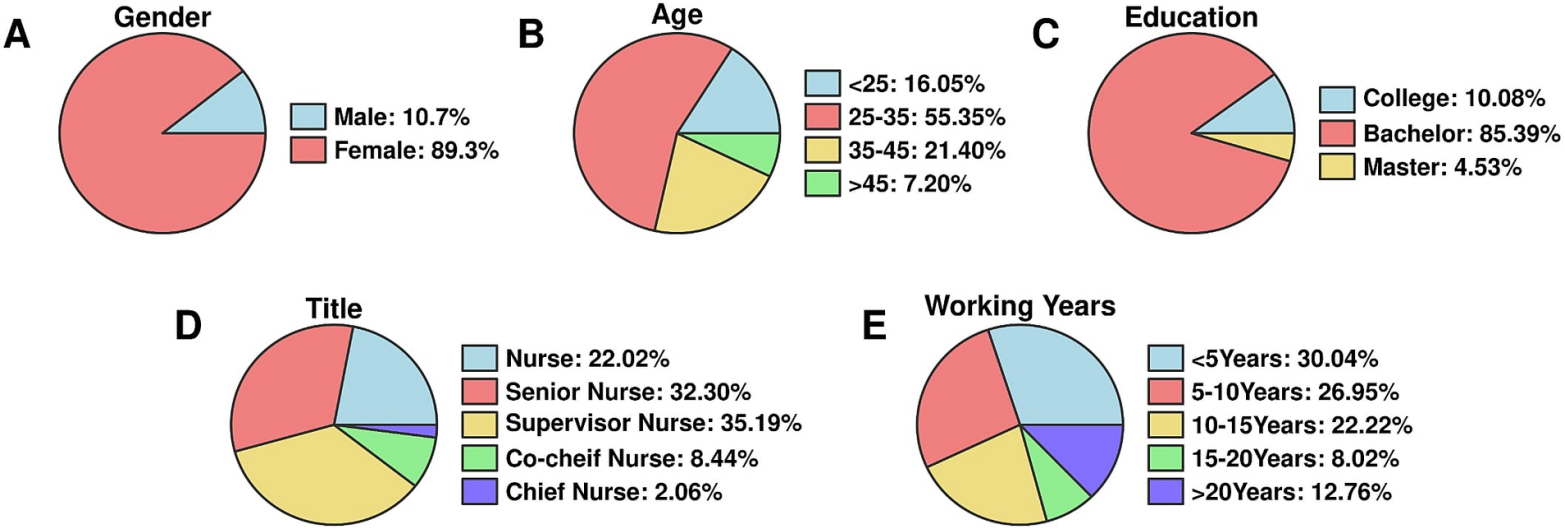
Demographic and professional characteristics of nursing survey respondents. **(A)** Gender distribution of nursing survey respondents, **(B)** age distribution of nursing survey respondents, **(C)** educational background of nursing survey respondents, **(D)** job position distribution of nursing survey respondents, **(E)** work experience distribution of nursing survey respondents.

The survey overwhelmingly consisted of female participants (89.3%, *n* = 434), with only 10.7% (*n* = 52) male respondents (Figure 1A). This gender imbalance may affect the representativeness of the data, and future efforts should aim to increase male participation through targeted recruitment strategies. The age distribution of the survey participants reveals a clear trend, with the majority of respondents falling within the 25–35 years age range (55.35%, *n* = 269), followed by the 35–45 years group (21.40%, *n* = 104), while those ≤25 years account for 16.05% (*n* = 78). The smallest group, representing 7.2% (*n* = 35), belongs to the ≥45 years category (Figure 1B). This data indicates that the majority of respondents are relatively young, predominantly in the 25–35 years bracket, while fewer nurses are found in the older age groups. The educational background data shows that the majority of participants hold a bachelor’s degree (85.39%, *n* = 415), while fewer hold a college diploma (10.08%, *n* = 49) or graduate-level education (4.53%, *n* = 22) (Figure 1C). This indicates that the overall educational level of participants is high, with a predominance of undergraduate qualifications. The majority of respondents held supervisory roles (35.19%, *n* = 171), followed by senior nurses (32.3%, *n* = 157). Staff nurses made up 22.02% (*n* = 107), while co-chief and chief nurses comprised 8.44% (*n* = 41) and 2.06% (*n* = 10), respectively (Figure 1D). This suggests a need to enhance development and training opportunities for senior nurses, as they represent a large proportion of the workforce. The highest proportion of respondents had <5 years of experience (30.04%, *n* = 148), followed by 5–10 years of experience (26.95%, *n* = 131). The smallest group had 15–20 years of experience (8.02%, *n* = 39) (Figure 1E). This highlights a relatively young workforce, which could imply high potential for innovation but also potential gaps in experience. It is recommended that mentorship and knowledge transfer programs be prioritized for less experienced staff to bridge these gaps. Chi-square tests and independent-samples *t*-tests were conducted to compare training needs across different demographic groups, including age, education level, and years of experience. Statistical significance was set at *p* < 0.05. Results showed that differences in training preferences across age groups and professional experience were statistically significant (*p* < 0.05). Specifically, younger nurses (≤35 years) expressed a stronger preference for Humanistic Innovation and Humanistic Education than older nurses. Additionally, senior nurses (≥10 years of experience) placed more emphasis on Humanistic Management and Humanistic Care modules, reflecting a greater need for leadership and care skills at more advanced levels of their careers.

### Training content preferences

**Figure 2:**
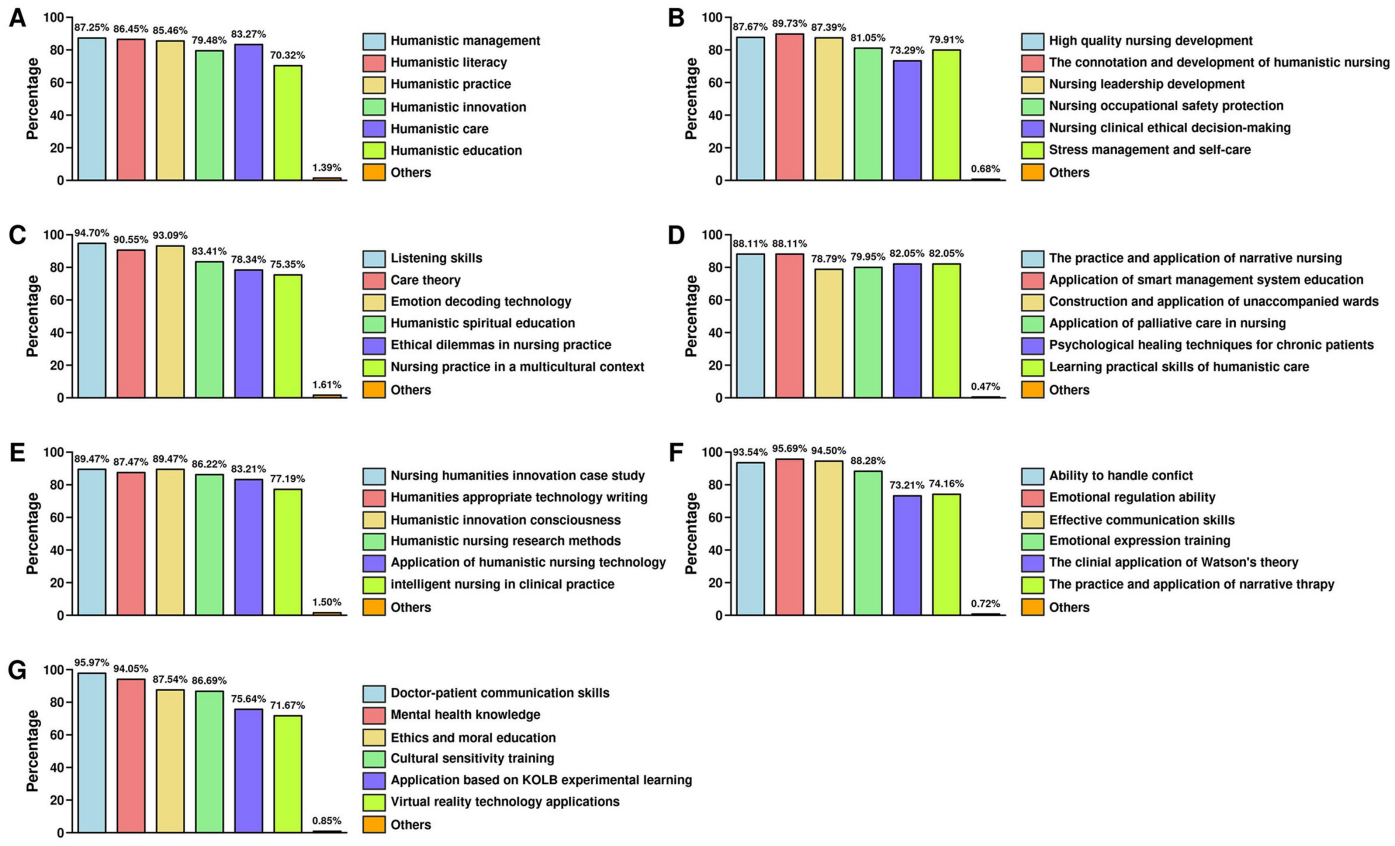
Course popularity and preferences across humanistic nursing training modules. **(A)** Training content demand in humanistic nursing system; **(B)** Course popularity in humanistic literacy training module; **(C)** Course popularity in humanistic nursing practice training; **(D)** Course preferences in the humanistic care module; **(E)** Course popularity in humanistic nursing innovation module; **(F)** Course preferences in the humanistic care module (skills-focused); **(G)** Course preferences in humanistic nursing education module.

The top six modules identified by participants for a systematic and structured humanistic nursing training program were Humanistic Management (87.04%), Humanistic Literacy (86.42%), Humanistic Practice (85.6%), Humanistic Care (83.33%), Humanistic Innovation (79.01%), and Humanistic Education (70.37%) (Figure 2A). These findings indicate particularly strong demand for management- and quality-focused modules, underscoring the importance of leadership and quality improvement in the nursing curriculum. By contrast, Humanistic Education received the lowest preference (70.37%), suggesting the need for more innovative teaching strategies to increase engagement. Furthermore, practical opportunities in Humanistic Practice should be expanded to ensure training is relevant to daily clinical work. Within Humanistic Management, the most highly valued courses included the connotation and development of humanistic nursing (89.73%), development of high-quality nursing (87.67%), and cultivation of nursing leadership (87.39%) (Figure 2B). Clinical ethical decision-making, although important, was relatively less favored (73.29%). In the Humanistic Literacy module, the most popular courses were listening skills (94.70%), emotional decoding techniques (93.09%), and caring theory (90.55%) (Figure 2C). Humanistic spirit education (83.41%), ethical dilemmas in practice (78.34%), and multicultural nursing (75.35%) were also valued but to a lesser extent. For Humanistic Practice, participants prioritized courses on narrative nursing and the effectiveness of smart management systems (88.11% each), followed by psychological healing for chronic illness and case-based learning of humanistic care (82.05%) (Figure 2D). Training on palliative care (79.95%) and unaccompanied ward development (78.79%) also received substantial interest, while very few participants selected “Other” options (0.47%).

In the Humanistic Innovation module, courses on innovation case studies and innovation awareness and practice attracted the greatest interest (89.47%), followed by writing humanistic appropriate technologies (87.47%) and research methods for humanistic nursing (86.22%) (Figure 2E). Interest in technology application in humanistic nursing (83.21%) and intelligent nursing systems (77.19%) suggests recognition of the role of technology, though these were comparatively less prioritized. For Humanistic Care, participants overwhelmingly favored interpersonal skill-based courses, including emotional regulation (95.69%), effective communication (94.50%), and conflict resolution (93.54%) (Figure 2F). Emotional expression (88.28%) was also popular, though slightly less so. By contrast, more theoretical courses such as Watson’s Caring Theory (73.21%) and narrative therapy (74.16%) received lower demand, reflecting participants’ preference for directly applicable, skills-based content. Finally, in the Humanistic Education module, the highest interest was expressed in doctor–patient communication (95.97%) and psychological health knowledge (94.05%), followed by ethics education (87.54%) and cultural sensitivity training (86.69%) (Figure 2G). Meanwhile, Kolb’s experiential learning (75.64%) and VR-based training (71.67%) were less favored, indicating that while innovative teaching methods are of some interest, foundational interpersonal and psychological skills are seen as more essential.

### Demographic comparisons (inferential analysis)

Chi-square and *t*-tests revealed statistically significant differences in training needs across demographic groups (*p* < 0.05). Younger nurses (≤35 years) expressed stronger interest in Humanistic Innovation and Humanistic Education, whereas senior nurses (≥10 years of experience) emphasized Humanistic Management and Humanistic Care. Nurses with <5 years of experience showed significantly higher demand for practice- and care-focused modules (*p* < 0.01). These results suggest that training curricula should be tailored by career stage, balancing innovation-focused content for younger nurses with leadership and care modules for senior staff.

### Preference for practice-oriented training

**Figure 3:**
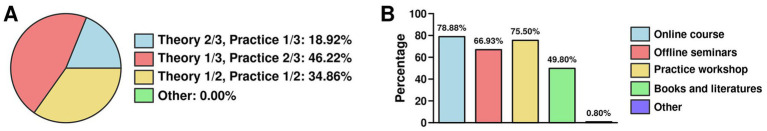
Preferences for training delivery methods and training focus in humanistic nursing. **(A)** Preference for training focus: theory vs. practice; **(B)** Preferred methods for delivering humanistic nursing system training.

Survey results also revealed a strong preference for practice-focused training in humanistic nursing. The option practice-focused, with practice accounting for 2/3 and theory for 1/3 was selected by the highest percentage of participants 46.3% *n* = 22, emphasizing the importance of hands-on experience in developing practical nursing skills. This was followed by the option equal balance between theory and practice by 34.86%, *n* = 169, suggesting that while participants value practical experience, they also recognize the importance of a balanced theoretical foundation. The least preferred option, “theory-focused, with theory accounting for 2/3 and practice for 1/3” 18.92% *n* = 92 (Figure 3A), reflects the participants’ preference for training that prioritizes real-world application over theoretical knowledge. These findings highlight a clear inclination toward training approaches that emphasize practical, experience-based learning, which is crucial for preparing nurses to meet the demands of modern healthcare settings.

Our survey results show that online courses were the most preferred method for delivering humanistic nursing system training, chosen by 78.88% *n* = 383 of participants, indicating a strong preference for flexible, accessible, and self-paced learning. Practical workshops also received significant support, with 75.50% *n* = 367 participants selecting this option, highlighting the participants’ desire for hands-on, experiential learning that allows them to directly apply their skills in real-world settings. In-person lectures were also favored by 66.93% (*n* = 325) of participants, emphasizing the continued value of face-to-face interactions for engaging with instructors and peers, as well as the opportunity for direct feedback and discussions. In contrast, books and materials received lower support, with only 49.80% of participants (*n* = 242) selecting this option (Figure 3B). This may reflect a decreased interest in traditional, text-based learning methods, with participants likely preferring more dynamic and interactive formats for training.

Statistical analyses confirmed that preferences for Humanistic Care and Humanistic Practice modules were significantly higher among nurses with less than 5 years of experience (*p* < 0.01), indicating the importance of practical training for newer nurses.

## Discussion

The importance of humanistic nursing in healthcare cannot be overstated. As the healthcare landscape continues to evolve, the role of nurses becomes increasingly pivotal in providing compassionate, patient-centered care (17). Our findings offer practical, data-driven insights into nurses’ training preferences across multiple domains of humanistic education, including preferred modules, delivery formats, and course content. Rather than presenting global generalizations, this discussion focuses on contextualizing these results within relevant educational frameworks and broader trends in nursing education.

Our study reveals that the majority of participants were female (89.3%) and relatively young, with most respondents between 25 and 35 years of age. This gender imbalance, although prevalent in the nursing profession, highlights a potential gap in male representation in nursing education and practice. Global studies, such as those by Lyu et al. (18) and Masibo et al. (19), have pointed out that nursing remains a predominantly female profession, with female nurses outnumbering male nurses significantly in most countries. While this reflects global workforce patterns, the limited male participation should be acknowledged as a factor affecting the diversity of perspectives captured in this survey. The emphasis on recruiting more male nurses into the profession is evident, and this issue also warrants further attention in humanistic nursing education. The educational background of our participants was relatively high, with 85.39% holding a bachelor’s degree. This aligns with the findings of global studies such as those by Kovner et al. and Kõrgemaa et al., which emphasize the increasing trend of higher education bachelor or masters among nursing professionals worldwide (20, 21). The relatively high academic profile of respondents may partially explain their strong interest in advanced modules, such as Humanistic Management and Innovation. The majority of respondents also held positions as Senior Nurses (32.3%) or supervise Nurses (35.19%), reflecting the current structure of nursing hierarchies. This is consistent with global nursing workforce trends, where a substantial portion of the workforce holds mid-level nursing titles (22). However, this distribution also suggests that further development of training programs for senior nurses is needed, as indicated in studies by Mlambo et al. (23). Regarding work experience, our findings indicated that most participants had less than 5 years of experience (30.04%), suggesting a relatively young workforce. This aligns with broader workforce challenges related to retention of experienced staff and underscores the value of structured mentorship programs to bridge knowledge and skills gaps between junior and senior nurses. This is which show that many countries face challenges in retaining experienced nurses. Our study highlights the importance of mentorship and knowledge transfer between seasoned nurses and newer entrants into the profession to bridge the experience gap and foster a more seasoned workforce. This can be achieved through structured mentorship programs, which are already being implemented in various parts of the world (24).

The core finding in this study revolves around the preferences for different training modules within humanistic nursing education. Participants overwhelmingly expressed interest in modules focusing on Humanistic Management (87.04%), Humanistic Quality (86.42%), and Humanistic Practice (85.6%). These preferences are suggestive of global trends toward integrating leadership, communication, and quality improvement content into nursing curricula, although the single-site design precludes definitive generalization (25). Studies have highlighted that incorporating leadership and management modules in nursing education can help equip nurses with the skills necessary to handle complex healthcare environments and improve patient outcomes (26, 27). Interestingly, while courses such as connotation and development of humanistic nursing received high demand (89.6%), other courses like clinical ethical decision-making in nursing (73.29%) were less popular. This suggests that while participants acknowledge the importance of ethics in nursing, there is a stronger preference for practical and quality improvement-focused content. A similar trend has been observed in global studies, which found that nurses prioritize practical, quality-related education over more theoretical, ethical-focused courses (28). However, ethical decision-making and stress management (which was also chosen by 79.91% of our respondents) must remain integrated into the curriculum as part of a well-rounded nursing education. In our study, the humanistic literacy module revealed a high demand for courses on listening skills (94.70%), caring theory (90.55%), and emotional decoding techniques (93.09%). These courses focus on soft skills, which are essential for fostering compassionate patient care. These findings are in line with global studies, such as those by Jahromi et al. (29), which stress the importance of communication and emotional intelligence in healthcare profession. Given this strong demand, curricula should prioritize structured modules on active communication and emotional intelligence, which are fundamental to patient-centered care. These soft skills have been shown to significantly enhance patient outcomes by improving nurse–patient interactions and fostering trust, which is a cornerstone of humanistic nursing. The popularity of courses like “The Practice and Application of Narrative Nursing” and “Effectiveness of Smart Management Systems in Education” in our study further highlights the participants’ interest in integrating technology with humanistic nursing practices. Technological advancements such as smart management systems can greatly enhance the efficiency of healthcare services while ensuring that humanistic care is not compromised (30).

Our study indicates a clear preference for practice-oriented training, with 46.3% of participants choosing the option that emphasizes 2/3 practice and 1/3 theory. This preference for hands-on learning aligns with studies emphasizing the growing demand for practical, experience-based education in nursing (31). As nursing practice becomes more complex, it is essential that training programs reflect real-world challenges. Many countries, including the U. S. and the U. K., have adopted a more practice-focused curriculum to better prepare nurses for clinical settings (32). Additionally, the preference for online courses (78.88%) and practical workshops (75.50%) demonstrates the growing trend toward flexible and interactive learning platforms in nursing education. This shift toward online and hybrid learning methods has been observed in global studies as well. Online education and workshops are gaining popularity as they offer flexible scheduling and foster increased interaction among participants, enhancing knowledge retention and its application in clinical practice (33).

Although this study was limited by its single-site convenience sample, predominantly female respondents, and cross-sectional design, these factors also highlight important directions for future work. The findings provide a valuable benchmark for understanding humanistic nursing training needs in a large tertiary hospital, while the gender imbalance reflects global workforce patterns and underscores the need to engage more male nurses in future research. Moreover, the cross-sectional nature of the study offers a timely snapshot of current training preferences, laying the groundwork for longitudinal studies to assess how such training impacts professional development and patient outcomes over time. Despite the absence of a formal power analysis, the robust sample size and strong survey reliability (Cronbach’s *α* = 0.85) provide a solid foundation for broader, multi-site investigations.

## Conclusion

This study identifies a clear need for structured, practice-oriented humanistic nursing education tailored to the preferences of nursing staff. The strong demand for modules in management, quality, and interpersonal skills, coupled with a preference for flexible and interactive delivery methods, provides actionable insights for curriculum development. However, given the single-site scope, demographic imbalance, and cross-sectional design, these results should be interpreted cautiously. Broader, multi-institutional studies with longitudinal follow-up are needed to confirm the impact of such training programs on both professional development and patient care outcomes. By addressing these challenges, future educational strategies can better equip nurses with the skills, empathy, and resilience necessary to advance patient-centered care in diverse healthcare contexts.

## Data Availability

The raw data supporting the conclusions of this article will be made available by the authors, without undue reservation.
